# Inflammatory and coagulatory parameters linked to survival in critically ill children with sepsis

**DOI:** 10.1186/s13613-018-0457-8

**Published:** 2018-11-16

**Authors:** Christian Niederwanger, Mirjam Bachler, Tobias Hell, Caroline Linhart, Andreas Entenmann, Agnes Balog, Katharina Auer, Petra Innerhofer

**Affiliations:** 1 Department of Pediatrics, Pediatrics I, Medical University of Innsbruck, Anichstrasse 35, 6020 Innsbruck, Austria; 2 Institute for Sports Medicine, Alpine Medicine and Health Tourism, UMIT - University for Health Sciences, Medical Informatics and Technology, Eduard Wallnöfer Zentrum 1, 6060 Hall in Tirol, Austria; 3 Department of Mathematics, Faculty of Mathematics, Computer Science and Physics, University of Innsbruck, Technikerstraße 13, 6020 Innsbruck, Austria; 4 Department of Medical Statistics, Informatics and Health Economics, Medical University of Innsbruck, Anichstrasse 35, 6020 Innsbruck, Austria; 5 Department of General and Surgical Critical Care Medicine, Medical University of Innsbruck, Anichstrasse 35, 6020 Innsbruck, Austria; 6 Department of Anaesthesiology and Critical Care Medicine, Medical University of Innsbruck, Anichstrasse 35, 6020 Innsbruck, Austria

**Keywords:** Fibrinogen, Sepsis, Children, Survival, Inflammation, Coagulation, Platelets

## Abstract

**Background:**

Sepsis is associated with a deflection of inflammatory and coagulative parameters, since some clotting factors are known to be involved in the host’s defense against infection and inflammation. These parameters could play a crucial role in the course of sepsis and be used as prognostic markers in critically ill children.

**Methods:**

A total of 250 critically ill pediatric patients diagnosed with sepsis were retrospectively analyzed to identify routinely measured predictors for in-hospital mortality at the peak level of C-reactive protein. Those parameters entered multivariate logistic regression analysis as well as a decision tree for survival.

**Results:**

Multivariate logistic regression analysis revealed fibrinogen, platelets and activated partial thromboplastin time (aPTT) at the peak level of C-reactive protein to be predictors for survival (*p* = 0.03, *p* = 0.01 and *p* = 0.02, respectively). An increase in fibrinogen and platelets is linked to survival, whereas an aPTT prolongation is associated with higher mortality; adjusted odds ratios (95% CI) for an increase of 100 mg/dl in fibrinogen are 1.35 (1.04–1.82) per 50 G/l platelets 1.94 (1.3–3.29) and 0.83 (0.69–0.96) for an aPTT prolongation of 10 s. Decision tree analysis shows that a fibrinogen level below 192 mg/dl (90.9% vs. 13% mortality) is most distinctive in non-survivors.

**Conclusions:**

High levels of fibrinogen and platelets as well as a non-overshooting aPTT are associated with a higher survival rate in pediatric patients with diagnosed sepsis. In particular, hypofibrinogenemia is distinctive for a high mortality rate in septic critically ill children.

**Electronic supplementary material:**

The online version of this article (10.1186/s13613-018-0457-8) contains supplementary material, which is available to authorized users.

## Introduction

Although the number of deaths caused by sepsis has drastically decreased in the last couple of decades [1], sepsis remains one of the main causes of mortality in infants and toddlers worldwide [1–3]. Sepsis in children peaks in the neonatal period and symptoms may be non-specific in those patients [4], while older children may show hyperthermia, tachycardia, tachypnea, hypotension and disorders in hemostasis up to the clinical picture of disseminated intravascular coagulation (DIC).

Sepsis is initially characterized by excessive production of pro-inflammatory cytokines, leukocyte activation and tissue damage, followed by release of anti-inflammatory cytokines, leukocyte deactivation and immunosuppression [5]. In the later phase of sepsis, compensatory release of anti-inflammatory molecules is thought to mediate a state of immunosuppression associated with significant impairment of immune cell function (immunoparalysis) [6].

The systemic inflammation during sepsis is observed by measuring leukocytes, procalcitonin, C-reactive protein and others. Leukocyte count is part of the definition of sepsis, but only at extreme levels are leukocytes associated with the progression of sepsis [7]. Data confirm that procalcitonin, which specifically increases in bacterial processes [8], is suitable as a diagnostic parameter in many cases in adults due to its high specificity [9, 10]. However, limited data are available for use in pediatric patients [11]. C-reactive protein reflects the inflammatory process and is widely used in clinical routine. Many studies have described an interrelation between an elevated C-reactive protein level and sepsis [12–15]. Thus, in clinical routine, daily C-reactive protein measurements might be used to assess the efficacy of treatment [16]. C-reactive protein can also be an indicator of organ failure [17–21] and therefore has potential for the surveillance of sepsis severity.

Systemic inflammation is followed by activation of the coagulation system, and conversely, components of the coagulation system significantly affect the inflammatory response [22]. This cross-link occurs on several levels: Pro-inflammatory cytokines stimulate the production of coagulation factors and coagulation factors mediate inflammation via binding to receptors of endothelium and immune cells [23]. The coagulation factors also play a direct role in host defense. For example, fibrinogen modulates the immune system [24, 25] and acts as a chemotactical factor for monocytes and neutrophils [26]. Furthermore, as part of the innate immune system, it limits bacterial spreading by forming fiber nets in which the pathogens become immobilized [24].

With few exceptions, such as antithrombin or platelets, the role of coagulation parameters in sepsis is largely ignored. New diagnostic, prognostic and therapeutic strategies can be deduced by observing coagulating laboratory data and, where appropriate, modifying them in the event of excessive activation or dysregulation of the system [27, 28]. An increase in the pro-coagulatory parameters to high level is seen rather negatively in the inflammatory process because of their pro-thrombotic potential [29], although elevation of the pro-coagulatory parameters might be beneficial during sepsis due to their role in host defense.

Therefore, it is worthwhile to examine the behavior and influence of coagulation parameters in combination with the typical inflammatory parameters during sepsis. Correct interpretation of these could facilitate assessment of the course of sepsis in critically ill children.

## Methods

This retrospective analysis comprises clinical data and routine laboratory parameters from 250 pediatric patients at the Pediatric Intensive Care Unit of Innsbruck Medical University Hospital.

### Inclusion of patients

All medical files of patients younger than 18 years of age who were treated at the pediatric intensive care unit (PICU) between January 1, 2000, and December 31, 2014, with the diagnosis of sepsis or systemic infection were reviewed. A total of 250 patients met the sepsis criteria of Goldstein [1]. There was no need to obtain oral and written informed consent from the study participants since the data were processed anonymously. The study protocol was approved by the institutional review board of the Medical University of Innsbruck (AN2013-0044).

### Data collection

We collected the demographic variables age, sex and the diagnosed underlying disease that triggered hospitalization. Septic shock was defined as the need for a vasoactive drug to maintain blood pressure in the normal range during the septic episode [1]. The C-reactive protein level was used to objectify the progression of sepsis, because it is an established parameter of sepsis [12–16] and is used in our clinic in children of all ages as opposed to IL-6 and procalcitonin.

The day on which C-reactive protein peaked was defined as day 0 in the report. The available routine parameters (fibrinogen, platelets, antithrombin, PT, aPTT, leukocytes) were observed from 3 days before until 3 days after day 0. In-hospital mortality was chosen as the outcome parameter.

To evaluate changes in therapy and outcome due to the protracted study period, we grouped patients into four time cohorts (2000–2004, 2005–2007, 2008–2010 and 2011–2014).

### Statistical analysis

A mathematician not involved in the study procedures or patient assessment (TH) was responsible for the statistical analyses using R, version 3.4.1. All statistical assessments were two sided, and a significance level of 5% was used. The hypothesis of a normal distribution was not reasonable for most of the continuous variables (Shapiro–Wilk normality test). The Wilcoxon rank sum test and Fisher’s exact test were applied to assess differences between survivors and non-survivors. We present continuous data as medians (25th–75th percentile) and binary variables as no./total no. (%). We show effect size and precision with estimated median differences between survivors and non-survivors for continuous data and odds ratios (OR) for binary variables, with 95% CIs.

Stratified by survival, the progression of inflammation- and coagulation-related parameters from 3 days prior to 3 days after the peak of C-reactive protein is illustrated by the sequence of the median with corresponding 95% CIs in a purely descriptive manner.

In the univariate analysis, significant predictors at the peak of C-reactive protein as well as the respective patient’s characteristics for survival were identified. Those variables entered a forward–backward stepwise selection by Akaike information criterion (AIC) to fit a logistic regression model for survival [30]. We provide adjusted odds ratios for the remaining predictors with 95% CIs. Receiver operating characteristic (ROC curves) analysis was performed, and ROC AUC is provided with 95% CIs. In addition, with the variables that entered the stepwise model selection, a recursive partitioning tree for survival was fitted using the R package rpart (version 4.1-11). All analyses were performed in a purely exploratory fashion.

## Results

### Patient characteristics

In total, 250 patients met the eligibility criteria for study inclusion and final analysis. Of those septic children, 41 (16.4%) did not survive while in hospital. Of the critically ill children, 53.2% suffered from sepsis and 26.4% from severe sepsis. Septic shock was reported in 51/250 (20.4%) children, of whom 21/51 (41.2%) died. Patients’ baseline characteristics stratified for survival and non-survival are presented in Table 1.

**Table 1:** Characteristics of patients stratified for survival and non-survival

Characteristics^a^	Total (*n* = 250)	Survivors (*n* = 209)	Non-survivors (*n* = 41)	Estimate with 95% CI^b^	*p* value^c^
Female gender	116/250 (46.4%)	97/209 (46.4%)	19/41 (46.3%)	1 (0.48 to 2.06)	1
Age^d^ (months)	34.83 (6.47–108.63)	35.5 (7.16–111.45)	23.8 (1.43–81.97)	3.33 (− 6.55 to 18.07)	0.2725
Neonates < 1 month	22/249 (8.8%)	14/208 (6.7%)	8/41 (19.5%)	3.34 (1.12 to 9.34)	0.0149
Infants 1–3 months	24/249 (9.6%)	20/208 (9.6%)	4/41 (9.8%)	1.02 (0.24 to 3.28)	1
*Diagnosed underlying disease*					
Central nervous system	46/250 (18.4%)	34/209 (16.3%)	12/41 (29.3%)	2.12 (0.89 to 4.82)	0.075
Cardiovascular	39/250 (15.6%)	31/209 (14.8%)	8/41 (19.5%)	1.39 (0.51 to 3.45)	0.4808
Digestive tract	41/250 (16.4%)	32/209 (15.3%)	9/41 (22%)	1.55 (0.59 to 3.74)	0.3546
Respiratory system	51/250 (20.4%)	41/209 (19.6%)	10/41 (24.4%)	1.32 (0.53 to 3.05)	0.5258
Oncologic	37/250 (14.8%)	31/209 (14.8%)	6/41 (14.6%)	0.98 (0.31 to 2.64)	1
Kidney	30/250 (12%)	22/209 (10.5%)	8/41 (19.5%)	2.05 (0.73 to 5.31)	0.1169
Liver	18/250 (7.2%)	15/209 (7.2%)	3/41 (7.3%)	1.02 (0.18 to 3.87)	1
Skin	11/250 (4.4%)	10/209 (4.8%)	1/41 (2.4%)	0.5 (0.01 to 3.68)	1
Other diagnosis	30/250 (12%)	25/209 (12%)	5/41 (12.2%)	1.02 (0.29 to 2.97)	1

^a^Binary data are presented as no./total no. (%) and continuous data as medians (25th–75th percentile)

^b^Odds ratio for binary variables and estimated median difference for continuous variables

^c^Differences between survivors and non-survivors assessed with Fisher’s exact test for binary variables and Wilcoxon rank sum test for continuous variables

^d^For one survivor, the exact age in months is not known

The population consisted of 134/250 (53.6%) male patients, and median (25th–75th percentile) age was 35 (6–109) months. The 22/249 (8.8%) neonates showed a significantly higher (*p* = 0.02) mortality rate, because 8/22 (36.4%) neonates as compared to 33/227 (14.5%) children older than 1 month did not survive the septic episode. The most commonly affected organ systems resulting in ICU admission were the respiratory system in 51/250 (20.4%) and the central nervous system in 46/250 (18.4%) children.

To rule out possible confounding due to the protracted study period (2000–2014), we grouped patients into four time cohorts: 80 patients were included in 2000–2004, 56 in 2005–2007, 68 in 2008–2010 and 46 in 2011–2014. Mortality was 12.5%, 28.6%, 13.2% and 13%, respectively, and was not significantly associated with the time periods (Fisher’s exact test: *p* = 0.07).

### Univariate analysis of parameters at the peak level of C-reactive protein

As presented in Table 2, no significant difference was observed in C-reactive protein or leukocyte levels between survivors and non-survivors at the peak level of C-reactive protein. There was a difference in the timing between hospital admission and C-reactive protein peak between survivors and non-survivors (*p* < 0.0001) with non-survivors having a longer time period to reach the C-reactive protein peak. In contrast, fibrinogen, platelets and antithrombin significantly differ. This is also the case for aPTT and PT.

**Table 2:** Parameters at the peak level of C-reactive protein stratified for survival and non-survival

Parameters^a^	Total (*n* = 250)	Survivors (*n* = 209)	Non-survivors (*n* = 41)	Estimate with 95% CI^b^	*p* value^c^	Not known^d^
C-reactive protein (mg/dl)	18.5 (9.8–28.4)	18.6 (10–28)	17.6 (7.9–31.9)	− 0.13 (− 5.3 to 4.55)	0.9491	0/0
Fibrinogen (mg/dl)	506 (329–675)	518 (383–685)	279 (173–514)	206 (118 to 289)	< 0.0001	56/9
Platelets (G/l)	124 (50–232)	147 (62.5–247)	63 (29–98)	74 (37 to 111)	< 0.0001	5/0
aPTT (s)	47 (38–56)	46 (36–53)	64 (53.5–83)	− 20 (− 28 to − 13)	< 0.0001	59/15
PT (%)	71 (51–84)	74 (55–85)	49 (36–71.5)	20 (10 to 30)	0.0001	59/14
Antithrombin (%)	63 (47–81)	65.5 (50–82)	51 (40–66)	14 (5 to 23)	0.0045	75/10
Leukocytes (G/l)	10.4 (4.6–17.8)	11.1 (5.3–17.5)	8.2 (3–20)	1.4 (− 1.6 to 5.1)	0.3342	4/5

^a^Data are presented as medians (25th–75th percentile)

^b^Estimated median difference

^c^Differences between survivors and non-survivors assessed with Wilcoxon rank sum test for continuous variables

^d^Number of missing measurements in survivors/non-survivors

### Progression of parameters around the peak level of C-reactive protein

**Fig. 1:**
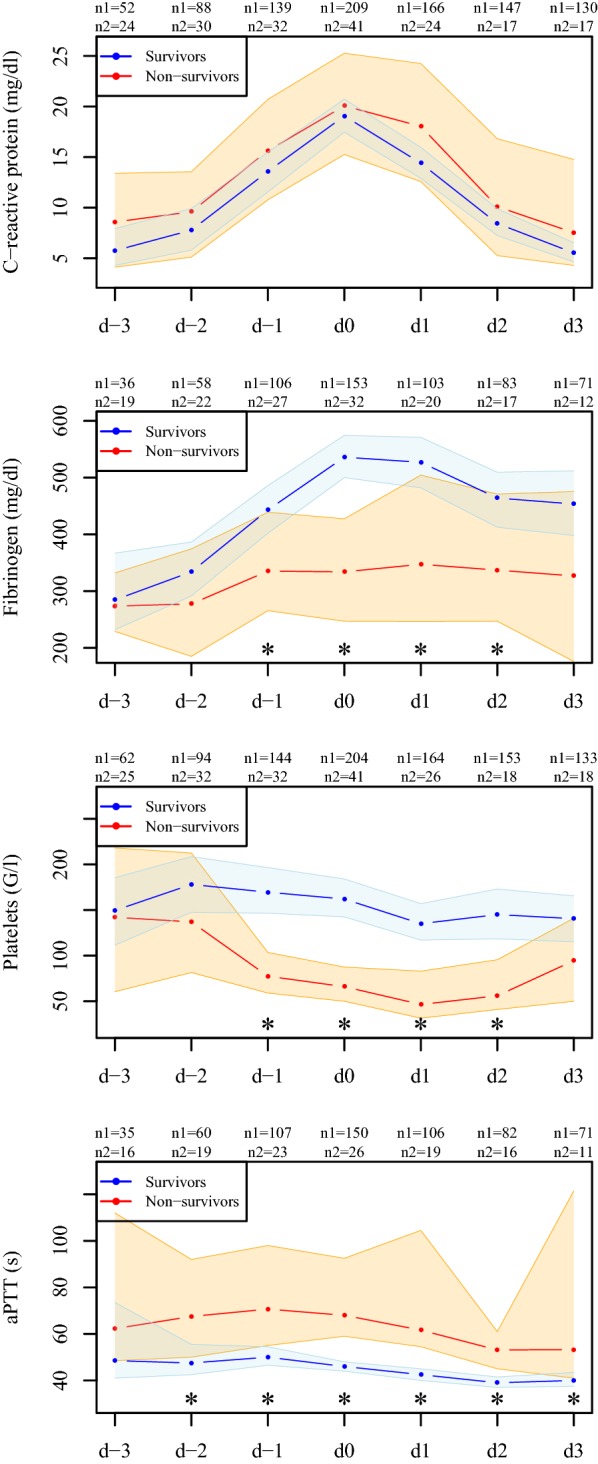
Progression of C-reactive protein and measured coagulation parameters. Depicted are medians with 95% CIs of the measured parameters for survivors (blue) and non-survivors (red) from 3 days prior to (d-3 to d-1) until 3 days after (d1 to d3) C-reactive protein peaked (d0). Asterisks indicate significant differences between survivors and non-survivors at the respective time point; *n*1 is the number of available measurements for survivors and *n*2 for non-survivors

**Fig. 2:**
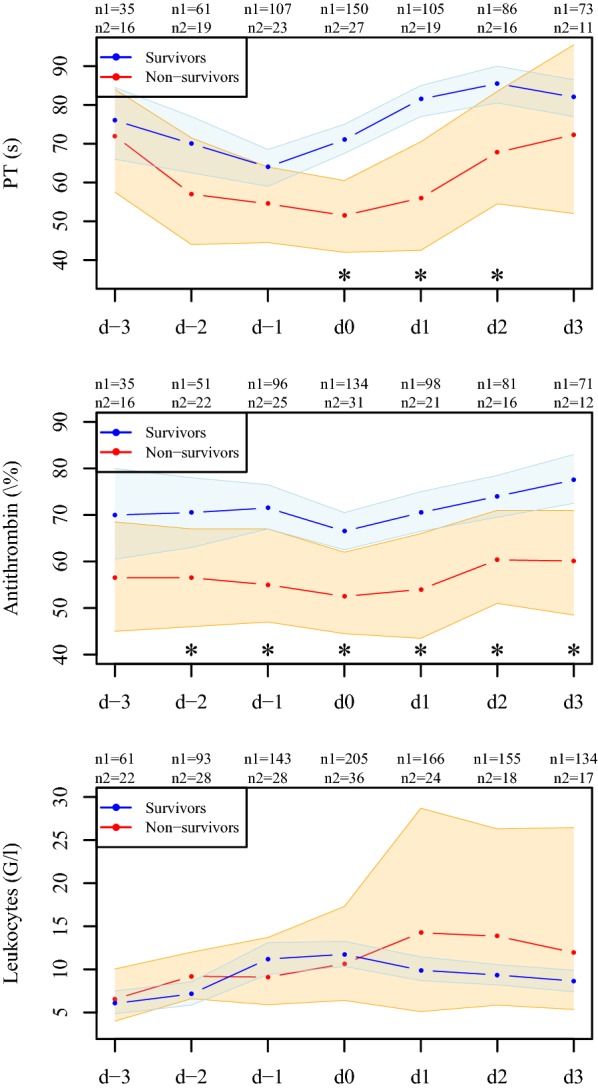
Progression of measured coagulation parameters. Depicted are medians with 95% CIs of the measured parameters for survivors (blue) and non-survivors (red) from 3 days prior to (d-3 to d-1) until 3 days after (d1 to d3) C-reactive protein peaked (d0). Asterisks indicate significant differences between survivors and non-survivors at the respective time point; *n*1 is the number of available measurements for survivors and *n*2 for non-survivors

The course of fibrinogen and antithrombin seems to be associated with the progression of C-reactive protein (Figs. 1, 2). Fibrinogen concentration followed the progression of C-reactive protein in survivors, but not in non-survivors. Note that for the main portion of the survivors, fibrinogen levels increased distinctly above the norm values around day 0.

Antithrombin levels were constantly higher in survivors than in non-survivors, but, in contrast to fibrinogen, irrespective of the progression of C-reactive protein. In survivors, platelets decreased slightly over time, while patients who did not survive showed a sharp decline in platelets already 2 days before the peak level of C-reactive protein was reached. Survivors showed significantly higher PT levels and shorter aPTT around the peak of C-reactive protein. Progression of leukocytes between survivors and non-survivors is comparable up to day 2.

CRP, fibrinogen, platelets, aPTT, PT and antithrombin levels were comparable between time cohorts (Kruskal–Wallis test: *p* = 0.40, *p* = 0.32, *p* = 0.58, *p* = 0.90, *p* = 0.17 and *p* = 0.22, respectively). Leukocyte count significantly differed between time cohorts (Kruskal–Wallis test: *p* = 0.04). Median leukocyte counts in G/l were 12.3 (6.13–20) in 2000–2004, 12.1 (6.8–17.1) in 2005–2007, 10.3 (3.1–16.4) in 2008–2010 and 7.7 (2.8–12) in 2011–2014.

### Multivariate logistic regression analysis for survival

The following significant univariate predictors for survival entered a stepwise model selection: fibrinogen, platelets, antithrombin, aPTT, PT and age. The resulting logistic regression model for survival includes fibrinogen, platelets and aPTT as variables, all of them being significant predictors for survival (*p* = 0.03, *p* = 0.01 and *p* = 0.02, respectively).

**Fig. 3:**
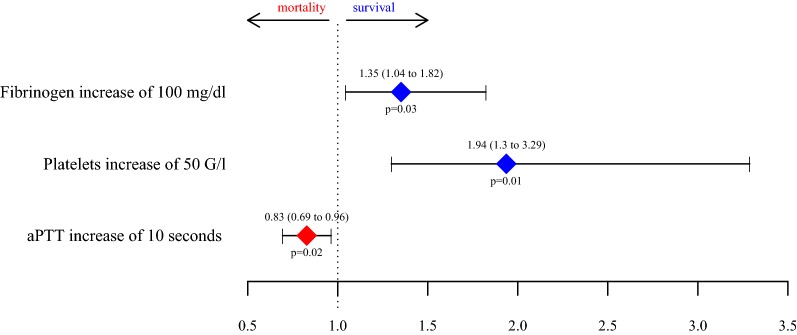
Adjusted odds ratios (95% CI) for survival retrieved from logistic regression. For an odds ratio greater than 1, an increase in the parameter decreases the mortality risk

Figure 3 shows the adjusted odds ratios (95% CI) retrieved from logistic regression. An increase in fibrinogen and platelets is linked to survival, whereas aPTT prolongation is associated with higher mortality. An increase of 100 mg/dl in fibrinogen increases the survival chance by 26%, per 50 G/l platelets by 48.4%, and aPTT prolongation of 10 s increases the mortality risk by 20.8%.

Adding the time cohort or the year of CRP peak as input variables resulted in the identical logistic regression model after stepwise model selection.

The ROC analysis (Additional file 1) for survival predicted by fibrinogen, platelets and aPTT resulted in an AUC of 0.74 (0.63–0.85), 0.71 (0.63–0.79) and 0.81 (0.73–0.90), respectively.

### Decision tree for survival

To gain a deeper insight into the interplay of coagulation parameters leading to death in septic children, a decision tree was fitted using the univariate predictors at the peak level of C-reactive protein and age to explore these interrelations.

**Fig. 4:**
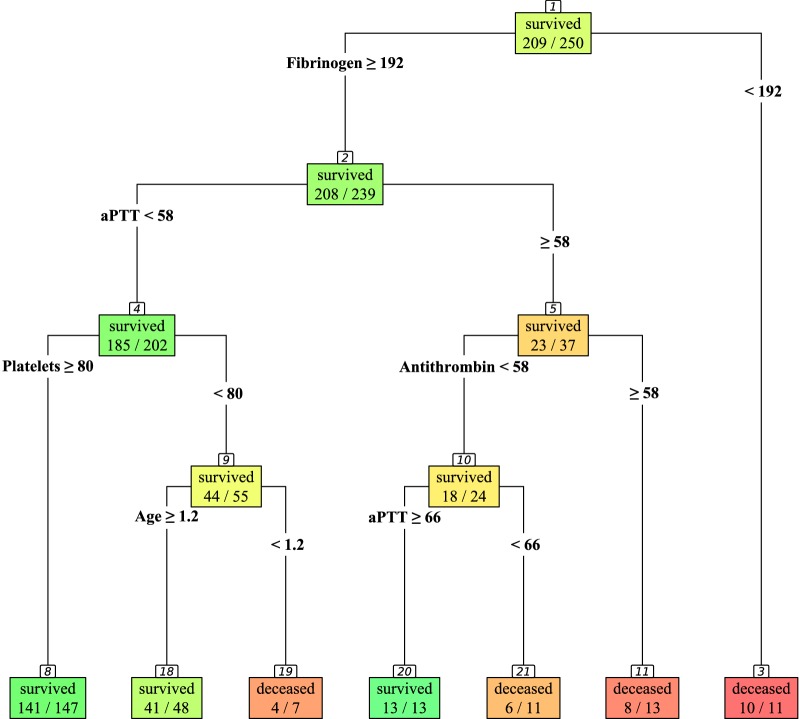
Decision tree for survival. The classification tree was fitted with univariate predictors at the peak of C-reactive protein and age

The obtained classification tree (see Fig. 4) shows that most distinctive in non-survivors is a fibrinogen level below 192 mg/dl [90.9% (58.7–99.8%) vs. 13% (8.1–18.5%) mortality]. For patients presenting with a fibrinogen level of at least 192 mg/dl, an aPTT of less than 58 s is associated with better outcome [8.4% (5.0–13.1%) vs. 37.8% (22.5–55.2%) mortality]. If, in addition, patients show a platelet count of at least 80 G/l, a low mortality rate is observed [4.1% (1.5–8.7%) vs. 20% (10.4–33.0%) mortality]. Patients with a platelet count below 80 G/l and younger than 1.2 months show high mortality as compared to older [57.1% (18.4–90.1%) vs. 14.6% (6.1–27.8%)].

In the case of a fibrinogen level of at least 192 mg/dl and an aPTT prolongation of 58 s or more, antithrombin levels below 58% led to lower mortality [25% (9.8–46.7%) vs. 61.5% (31.6–86.1%)], especially in patients exceeding an aPTT of 66 s [0% (0.0–24.7%) vs. 54.5% (23.4–83.3%) mortality].

The identical decision tree was obtained when the time cohort or the year of C-reactive protein peak was added as predictors for survival, indicating that there was no confounding due to the protracted study period (2000–2014).

In order to predict mortality with fibrinogen < 192 mg/dl, platelets < 80 G/l and aPTT > 58 s, sensitivity and specificity were calculated (Additional file 2).

### Age-adjusted hypofibrinogenemia

The fibrinogen level of each child was classified as hypo-, normo- or hyperfibrinogenemia according to the age-dependent norm value ranges. For normal fibrinogen values, please see the table in Additional file 3.

**Fig. 5:**
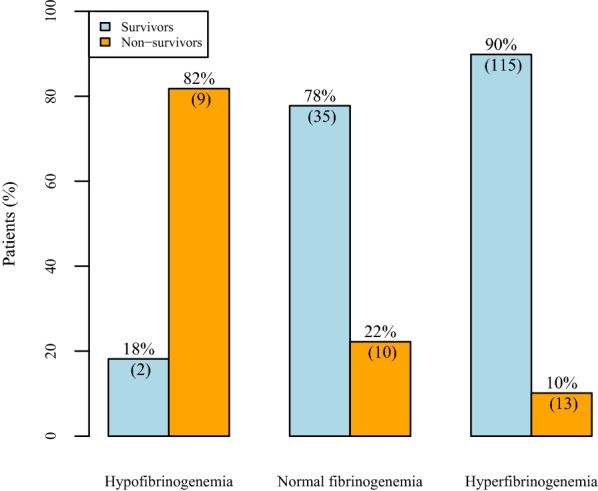
Survival by age-adjusted hypofibrinogenemia, normal fibrinogenemia and hyperfibrinogenemia. The classification is based on the age-adjusted norm value ranges

Patients presenting with hypofibrinogenemia have a significantly higher mortality rate than do patients with normo- or hyperfibrinogenemia: OR 28.42 (5.42–284.81), *p* < 0.0001. As depicted in Fig. 5, 82% of the patients with low fibrinogen levels died, whereas the mortality rate in patients with fibrinogen levels within the normal range was 22% and 10% in children with an elevated fibrinogen level.

No significant difference in the occurrence of a diagnosed thromboembolic event was observed for the 5/128 (3.9%) children with hyperfibrinogenemia as compared to the 4/56 (7.1%) patients with fibrinogen within the normal values or below: OR 0.53 (0.11–2.78), *p* = 0.46.

## Discussion

In this study, we investigated the impact of routinely measured coagulation and inflammation parameters on in-hospital survival in pediatric patients diagnosed with sepsis. The main result is that C-reactive protein at its peak level does not significantly differ between survivors and non-survivors, whereas fibrinogen, platelets and aPTT are predictors of survival. An increase in fibrinogen and platelets is linked to survival, whereas aPTT prolongation is associated with higher mortality. Most distinctive for mortality are fibrinogen levels below 192 mg/dl. Especially in children younger than 1.2 months, isolated thrombocytopenia is associated with poor outcome. In general, age-adjusted hypofibrinogenemia is significantly associated with a poor chance of survival.

In some points, these results stand in contrast to findings reported in the literature. Several studies claim that C-reactive protein is of prognostic value in sepsis [12–15]. However, in our study focusing on mortality, C-reactive protein levels did not allow any differentiation between survivors and non-survivors during the observation period of 7 days. Most likely, in children as well as in adults with diagnosed sepsis C-reactive protein levels are highly elevated without predicting final outcome.

Among the variables routinely measured in our study at the peak level of C-reactive protein, multivariate analysis revealed that only three of these coagulation parameters are essential for the prediction of survival in septic children: An increase in fibrinogen and platelets lowers the mortality risk, while a prolongation of aPTT increases the risk.

We found that hypofibrinogenemia is associated with very high mortality. This is in accordance with Esroy et al., who also confirm in their study that deceased patients exhibited significantly lower fibrinogen levels than did survivors [31]. Moreover, in baboons, survivors had higher fibrinogen levels than did non-survivors after induced sepsis [32]. This was also found in several earlier studies with fibrinogen-deficient mice following induced sepsis [33, 34].

The reason why fibrinogen is associated with survival in sepsis could be that it helps the immune system limit bacterial growth and enhance bacterial clearance [35]. The fibrin net captures and immobilizes invasive bacteria [24], thus restricting local spreading [24, 36]. Once the fibrinolysis sets in, plasminogen releases fibrinogen-derived so-called antimicrobial peptides (AMPs), thus causing an antimicrobial environment to arise within the clot. Such a peptide is the Bß15–42 fragment, and an unambiguous antimicrobial effect of this protein was already proven by S. aureus, group A Streptococcus (GAS) and group B Streptococcus (GBS) [25]. Besides that, this peptide binds to the VE cadherin of the endothelial cells and thus reinforces the tight junctions, which has a positive effect on organ failure and survival during sepsis [37, 38]. The importance of fibrinogen, fibrinolysis and the consequently released peptides during sepsis and their beneficial impact on infection, multiple-organ dysfunction and reduced mortality have already been proven in several studies [39–41].

In our study, age-adjusted hyperfibrinogenemia is associated with increased survival in sepsis. In studies of adult patients, this connection between acute and transient fibrinogen levels in the context of sepsis and survival was also observed [42]. In this context of high levels of pro-thrombotic factors, the fear of a hypercoagulatory state during sepsis is present and justified, since this could contribute to the development of thrombosis. In our study, the incidence rate of thromboembolic events was not significantly increased in patients with hyperfibrinogenemia.

Low fibrinogen levels may reflect ongoing consumption and deposition, development of DIC and MODS [43, 44]. Sepsis itself also causes severe damage to the liver, via hemodynamic changes as well as the direct or indirect destruction of hepatocytes or both [45]. The destroyed hepatocytes are no longer able to synthesize a sufficient amount of fibrinogen, which might be another reason for the low fibrinogen levels in non-survivors.

High concentrations of fibrinogen before the C-reactive protein peak in our study rarely coincided with platelet concentrations below the critical limit of 100 G/l. Low platelets in addition to low fibrinogen levels result in weak clot firmness, which is known to lead to poor outcome [4, 46]. Especially in neonates, isolated thrombocytopenia leads to increased mortality. This may be explained by the fact that during the first 6 weeks after birth, the plasmatic coagulation cascade is not fully developed and therefore hemostatic competence heavily relies on platelet function [47].

Overall, a decreased platelet count is associated with mortality. This might be due to the fact that thrombocytes are also an important part of the innate immune system. Whenever pathogens are present, platelets bind to them via the glycoprotein receptor IIb/IIIa and Toll-like receptors. That either happens directly, or it is mediated by plasma proteins like fibrinogen [48]. The activated platelets release antimicrobial peptides like defensins, kinocidins and thrombocidins to kill bacteria. In addition, they release members of the chemokine family like platelet factor 4 (PF4, CXCL-4) in order to attract other immune cells [49, 50].

The third coagulation parameter essential for the prediction of mortality in septic children in our study was aPTT. Prolongation leads to poorer outcome. A long aPTT might reflect the most severe cases of sepsis due to consumption of coagulation factors or high-dose heparin therapy, but may also be caused by a FXII deficiency. In septic patients, FXII deficiency can be protective via attenuation of the FXII-dependent bradykinin generation, complement activation and further contact pathway activation. This consequence of low FXII levels might explain why 13/13 children with fibrinogen levels above 192 mg/dl and an aPTT above 66 s survived, despite an antithrombin level below 58.

Compared with developed countries, at 16.4% the overall mortality of our children was in the upper frame [51, 52]. Due to the tertiary care status of our university hospital, the study included a large number of seriously ill children. Of them, 20.4% developed septic shock with a mortality rate of 41.2%. The literature reports a mortality rate of 20–30% in the presence of septic shock [53, 54], which increases to 52% in the case of additional MODS.

The limitations of our study have to be mentioned. The retrospective design of the study precludes assessment of causal relationships. Nevertheless, the main finding that high fibrinogen levels are beneficial in sepsis can be well explained by recently reported pathophysiological mechanisms. Further, not all the considered parameters were available throughout the observation period of 7 days. Especially for children who died rapidly or were transferred to another hospital soon after C-reactive protein peaked, data are lacking and a bias could be present in the consecutive days due to the smaller sample size. Therefore, the main analyses were performed solely on the day of C-reactive protein peak. The number of 250 sepsis cases diagnosed in children over a period of 14 years could be considered low. A deeper insight might be gained in a prospective setting.

Interpretation of the results of the multivariate logistic regression analysis for survival and decision tree analysis should be done carefully, since it cannot be concluded that these coagulation parameters are independently associated with survival because major well-known risk factors for death such as organ dysfunction or lactate were not taken into account. These analyses were used solely in an explorative fashion to describe the relation of the inflammatory and coagulatory parameters to survival. Therefore, this study is mainly descriptive and cannot determine the predictive value of coagulation parameters for outcome after sepsis. Another limitation is the fact that some values, such as low fibrinogen or low platelets, might have led to interventions that were not captured by this retrospective study. This could have influenced the magnitude of the associations. Despite all limitations, this study gives new insight into the course of sepsis and provides a good basis for a prospective study using these parameters as proxy for mortality, like organ dysfunction resolution or new and progressive MODS.

## Conclusion

The link between inflammation and coagulation plays a crucial role in children with sepsis. C-reactive protein does not allow discrimination between survivors and non-survivors. In contrast, increased levels of fibrinogen and platelets are linked to survival and distinctively reflect the inflammatory process. Prolonged aPTT is associated with lower survival, which might reflect therapy-related measures needed due to disease severity.

## Additional files

**Additional file 1.** Receiver operating characteristic (ROC) curves and area under the ROC curve (AUC) values for survival. The ROC curves showing the predictive value of fibrinogen. (A) Platelets (B) and aPTT (C). ROC AUC is provided with 95% CIs.

**Additional file 2.** Sensitivity and specificity analysis for mortality of fibrinogen, platelets and aPTT.

**Additional file 3.** Fibrinogen (Clauss method) norm value ranges for female and male children.

## Abbreviations

AIC: Akaike information criterion
AMP: antimicrobial peptides
aPTT: activated partial thromboplastin time
CRP: C-reactive protein
DIC: disseminated intravascular coagulopathy
FXII: coagulation factor XII
GAS: group A Streptococci
GBS: group B Streptococci
ICD: International Statistical Classification of Diseases and Related Health Problems
MODS: multiple organ dysfunction syndrome
OR: odds ratio
PF4: platelet factor 4
PICU: pediatric intensive care unit
PT: prothrombin time
