# Supplementary material for: Inflammatory and coagulatory parameters linked to survival in critically ill children with sepsis
Source: Ann Intensive Care. 2018 Nov 16;8:111. doi: 10.1186/s13613-018-0457-8 (PMC6240023; doi:10.1186/s13613-018-0457-8)
Supplement: Supplementary file 1 — Additional file 1. Receiver operating characteristic (ROC) curves and area under the ROC curve (AUC) values for survival. The ROC curves showing the predictive value of fibrinogen. (A) Platelets (B) and aPTT (C). ROC AUC is provided with 95% CIs. [file 13613_2018_457_MOESM1_ESM.pdf]

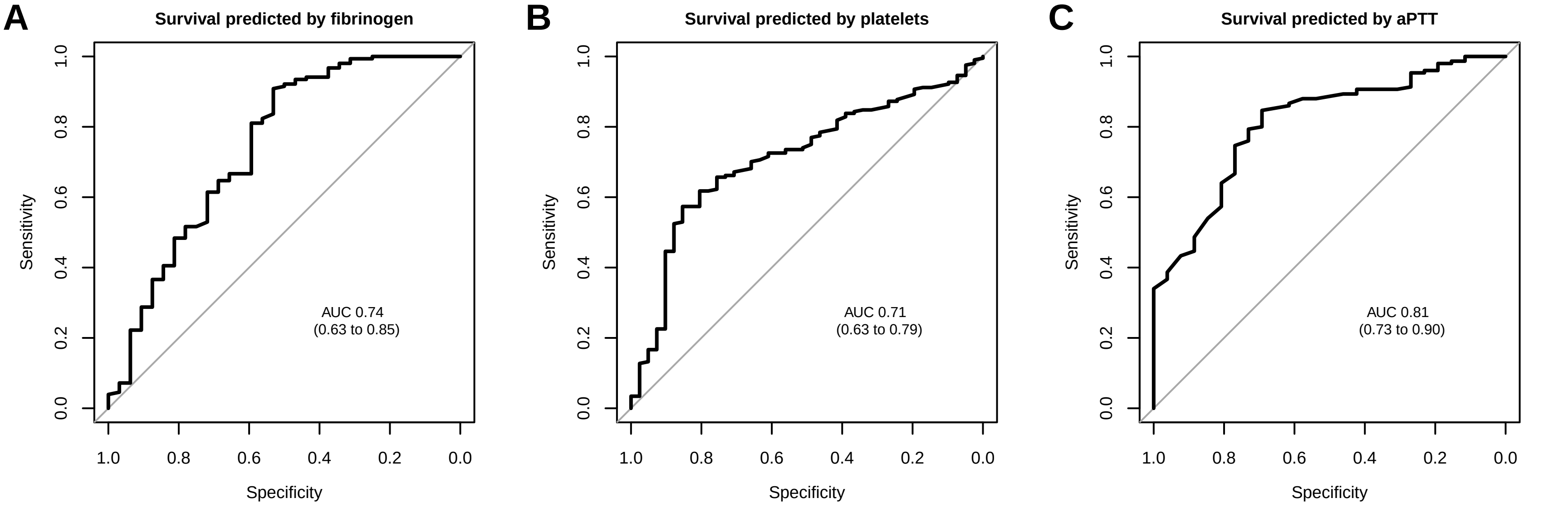
A
Survival predicted by fibrinogen
AUC 0.74
(0.63 to 0.85)
B
Survival predicted by platelets
AUC 0.71
(0.63 to 0.79)
C
Survival predicted by aPTT
AUC 0.81
(0.73 to 0.90)
Sensitivity
Specificity
0.0
0.2
0.4
0.6
0.8
1.0
