# Supplementary material for: Inflammatory and coagulatory parameters linked to survival in critically ill children with sepsis
Source: Ann Intensive Care. 2018 Nov 16;8:111. doi: 10.1186/s13613-018-0457-8 (PMC6240023; doi:10.1186/s13613-018-0457-8)
Supplement: Supplementary file 2 — Additional file 2. Sensitivity and specificity analysis for mortality of fibrinogen, platelets and aPTT. [file 13613_2018_457_MOESM2_ESM.docx]

***Additional file 3****. Sensitivity and Specificity analysis for mortality of fibrinogen, platelets and aPTT.*

|  | **Sensitivity** | **Specificity** |
| --- | --- | --- |
| Fibrinogen < 192 mg/dl | 0.99 | 0.31 |
| Platelets < 80 G/l | 0.70 | 0.66 |
| aPTT > 58 sec | 0.85 | 0.69 |
