# Supplementary material for: Inflammatory and coagulatory parameters linked to survival in critically ill children with sepsis
Source: Ann Intensive Care. 2018 Nov 16;8:111. doi: 10.1186/s13613-018-0457-8 (PMC6240023; doi:10.1186/s13613-018-0457-8)
Supplement: Supplementary file 3 — Additional file 3. Fibrinogen (Clauss method) norm value ranges for female and male children. [file 13613_2018_457_MOESM3_ESM.docx]

***Additional file 1****. Fibrinogen (Clauss method) norm value ranges for female and male children.*

| **Age** | | **Fibrinogen (mg/dl)** | | |
| --- | --- | --- | --- | --- |
| < 3 | months | 159.00 | - | 330.00 |
| ≥ 3 and < 6 | months | 183.00 | - | 398.00 |
| ≥ 6 and < 12 | months | 202.00 | - | 426.00 |
| ≥ 1 and < 4 | years | 214.00 | - | 396.00 |
| ≥ 4 and < 9 | years | 216.00 | - | 379.00 |
| ≥ 9 and < 18 | years | 223.00 | - | 418.00 |
